# Concerted evolution of duplicated mitochondrial control regions in three related seabird species

**DOI:** 10.1186/1471-2148-10-14

**Published:** 2010-01-14

**Authors:** James A Morris-Pocock, Scott A Taylor, Tim P Birt, Vicki L Friesen

**Affiliations:** 1 Department of Biology, Queen's University, Kingston, Ontario K7L 3N6, Canada

## Abstract

**Background:**

Many population genetic and phylogenetic analyses of mitochondrial DNA (mtDNA) assume that mitochondrial genomes do not undergo recombination. Recently, concerted evolution of duplicated mitochondrial control regions has been documented in a range of taxa. Although the molecular mechanism that facilitates concerted evolution is unknown, all proposed mechanisms involve mtDNA recombination.

**Results:**

Here, we document a duplication of a large region (cytochrome *b*, tRNA^Thr^, tRNA^Pro^, ND6, tRNA^Glu ^and the control region) in the mitochondrial genome of three related seabird species. To investigate the evolution of duplicate control regions, we sequenced both control region copies (CR1 and CR2) from 21 brown (*Sula leucogaster*), 21 red-footed (*S. sula*) and 21 blue-footed boobies (*S. nebouxii*). Phylogenetic analysis suggested that the duplicated control regions are predominantly evolving in concert; however, approximately 51 base pairs at the 5' end of CR1 and CR2 exhibited a discordant phylogenetic signal and appeared to be evolving independently.

**Conclusions:**

Both the structure of the duplicated region and the conflicting phylogenetic signals are remarkably similar to a pattern found in *Thalassarche *albatrosses, which are united with boobies in a large clade that includes all procellariiform and most pelecaniform seabirds. Therefore we suggest that concerted evolution of duplicated control regions either is taxonomically widespread within seabirds, or that it has evolved many times.

## Background

Concerted evolution of nuclear gene families is well documented and recognized as a fundamental force that influences genetic variation at many nuclear loci [1]. However, concerted evolution of mitochondrial DNA (mtDNA) has received much less attention. The gene content of vertebrate mitochondrial genomes is highly conserved and consists of 37 genes (13 protein coding genes, two rRNA genes and 22 tRNA genes) and a non-coding control region involved in the initiation of transcription and replication [2]. While this same suite of genes is present in all vertebrate mitochondrial genomes, gene order is highly variable. For example, at least four common gene orders exist for birds alone [3]. Additionally, many avian mitochondrial genomes possess two copies of some genes due to gene duplication (e.g., [4,5]). Traditional theory suggests that mtDNA gene duplication followed by deletion or degeneracy of one of the duplicated copies may lead to mtDNA gene rearrangements [2]. Degenerate copies of mitochondrial genes and non-coding regions have been found in many taxa, including at least four orders of birds [6]. However, duplicated mtDNA genes and non-coding regions have also been found where both copies appear functional (see [4,5] for examples in birds). Remarkably, in some vertebrate mtDNA genomes where the non-coding control region has been duplicated, both copies appear functional and extreme sequence similarity between the copies suggests that the two control regions are evolving in concert (e.g., [7]).

Although concerted evolution of vertebrate duplicated mtDNA regions is becoming increasingly documented, a number of important questions remain. First, the taxonomic extent of concerted evolution of duplicated regions is not well understood within birds. Concerted evolution of duplicated control regions has been documented in both *Amazona *parrots and *Thalassarche *albatrosses [4,5], even though parrots and albatrosses are phylogenetically very divergent [8]. It remains to be seen whether other avian species closely related to either albatrosses or parrots also have duplicate control regions that evolve in concert. Second, the implications of mtDNA concerted evolution for phylogeographic and phylogenetic research are still unclear. If mtDNA regions typically used for phylogeographic research are often duplicated and evolve in concert, then particular care may be needed to amplify homologous regions in all individuals in a study. On the other hand, Tatarenkov and Avise [7] documented that the rate of gene conversion responsible for concerted evolution of duplicated mitochondrial control regions of mangrove killifish (*Kryptolebias marmoratus*) was extremely fast compared to the nucleotide substitution rate within the control regions. Therefore, duplicated copies within individuals were more closely related to each other than either was to copies from other individuals. In cases like this, amplification of non-homologous regions should not seriously affect phylogeographic inference. Third, although many studies have investigated concerted evolution of mitochondrial control regions, few have investigated evolution of duplicated regions adjacent to the control region. Knowledge of concerted evolution in these regions may be of particular importance because many genes adjacent to the control region (e.g., cytochrome *b*, ND6, 12S rRNA) are used in population genetic and phylogenetic research. Moreover, the pattern of sequence evolution around duplicated control regions may provide insight into the molecular mechanism responsible for concerted evolution of duplicated mtDNA.

The Sulidae is a small family of seabirds that consists of seven booby species and three gannet species [9,10]. The most recent avian molecular phylogeny, estimated from 19 nuclear loci, unites the Sulidae in a large clade that contains all other water birds and importantly, the albatrosses (Aves: Diomedeidae; [8]). Here we present evidence of a large duplication of the mitochondrial genome in the evolutionary history of the Sulidae. We investigate the evolution of the duplicated regions in three species: brown (*Sula leucogaster*), red-footed (*S. sula*) and blue-footed boobies (*S. nebouxii*). Finally, we discuss the implications of animal mtDNA concerted evolution in the context of traditional phylogeographic studies.

## Methods

### Sample collection

We obtained tissue samples from 21 brown, 21 red-footed and 21 blue-footed boobies from throughout their breeding ranges (Table 1). Brown booby samples from Johnston Atoll and red-footed booby samples from Johnston Atoll, North Keeling Island and Aldabra Atoll consisted of growing feathers collected from chicks caught at nests. All other samples consisted of blood taken from adults or chicks caught at nests. We extracted DNA using either a standard phenol/chloroform technique [11] or the DNeasy® tissue kit, following the manufacturer's protocol (Qiagen, Mississauaga).

**Table 1:** Sampling site locations and numbers of individuals sampled (N)

Species	Ocean Basin	Colony	Abbreviation	Latitude	Longitude	N
Brown booby (br)	Pacific	Palmyra Atoll	Pal	05°33' N	162°50' W	3
		Johnston Atoll	Jon	16°45' N	169°31' W	3
		Farralon de San Ignacio	Fsi	25°24' N	108°50' W	3
		Piedra Blanca	Pbl	21°25' N	106°28' W	2
		Isla San Benedicto	Sbe	19°19' N	110°49' W	1
	Atlantic	Isla Monito	Mon	18°05' N	67°53' W	3
		Cape Verde	Cvd	15°05' N	24°48' W	3
		Ascension	Asn	7°56' S	14°22' W	3
Red-footed booby (rf)	Pacific	Genovesa, Galapagos	Gen	00°20' N	89°57' W	3
		Palmyra Atoll	Pal	05°33' N	162°50' W	3
		Tern Is.	Trn	23°52' N	166°17' W	3
	Atlantic	Isla Monito	Mon	10°18' N	109°13' W	3
		Ascension	Asn	7°56' S	14°22' W	1
	Indian	Europa Is.	Eur	22°22' S	40°22' E	2
		Aldabra Atoll	Ald	09°24' S	46°22' E	3
		North Keeling Is. (Cocos)	Coc	12°07' S	96°54' E	3
Blue-footed booby (bf)	Pacific	Champion Island, Galapagos	Cha	1°13' S	90°21' W	2
		Espanola, Galapagos	Esp	1°21' S	89°41' W	2
		Seymour Island, Galapagos	Sey	0°23' S	90°17' W	3
		El Rancho, Mexico	Elr	25°06' N	108°22' W	2
		Farralon de San Ignacio	Fsi	25°24' N	108°50' W	3
		Lobos de Tierra	Ldt	6°26' S	80 °51' W	3
		La Plata	Lpl	1°16' S	81°03' W	2
		Islas Marietas	Mar	21°33' N	106°23' W	2
		Isla San Ildefonso	San	26°43' N	111°29' W	2
**Total**						**63**

### Laboratory methods

Initial attempts to amplify and sequence the mitochondrial control region in all three species using conserved avian primers failed to produce unambiguous sequence. Specifically, while PCR products yielded clear sequence at the 3' end of the control region, superimposed peaks of two nucleotides at single sites were consistently present in chromatograms at the 5' end, rendering accurate base calling impossible. Among other possibilities (see Results), the presence of a duplicated control region was suspected. Therefore we used long-template PCR (LT-PCR; Expand Long Template PCR System®, Roche Applied Science, Manheim, Germany) to verify the presence and investigate the structure of potentially duplicated control regions. All long template PCR reactions were performed in 50 μL reactions containing 1 unit of 1× Expand Long Template PCR Buffer 1, 350 μM each of the four dNTPs, 800 nM each of the heavy and light strand primers, 3.75 units of Expand Long Template Enzyme Mix and approximately 150 nanograms of DNA template. LT-PCR was performed with two minutes initial denaturation at 94°C, followed by 25 cycles of 94°C for 10 seconds, 50-60°C for 30 seconds and 72°C for greater than four minutes (extension for the first 10 cycles was 4 minutes then an additional 20 seconds were added to the extension time every cycle), followed by a final extension for 7 minutes at 72°C.

**Figure 1:**
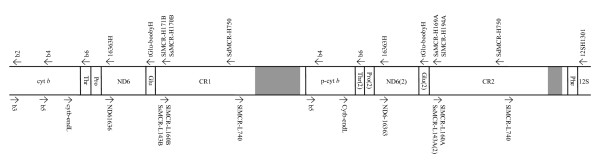
**Location of PCR primers within the duplication region of the sulid mitochondrial genome**. Schematic representation of the duplicated portion of the sulid mitochondrial genome, and approximate locations of PCR primers. L-strand primers are listed underneath the schematic, while H-strand primers are listed above. Gene abbreviations are as follows: cyt *b *= cytochrome *b*, ND6 = NADH dehydrogenase subunit 6, 12S = 12SrRNA, CR1 and CR2 = control region 1 and control region 2, Thr = tRNA-Threonine, Pro = tRNA-Proline, Glu = tRNA-Glutamic acid, Phe = tRNA-Phenylalanine. Grey areas correspond to long repetitive regions in Domain III of the control regions that could not be sequenced completely. All primer sequences are given in Table 2.

In one individual of each species, we amplified the region between the two presumed control regions using an unconventional primer pairing (SdMCR-H750 and SlMCR-L740, source of all primers listed in Table 2). These primers were oriented such that they would only amplify a product if duplicate control regions existed (i.e., if only a single control region existed, no product would be amplified under standard PCR conditions; Figure 1). This amplification yielded products of approximately 4000, 3000 and 2800 base pairs in brown, red-footed and blue-footed boobies, respectively. We sequenced both strands of these amplification products using the original amplification primers and a suite of internal primers (Figure 1, Table 2). Although some portions could not be sequenced reliably due to complex repeat regions (Figure 1, grey areas; see Results), all other parts were sequenced with at least two different primers on each strand. Sequencing was performed using either (1) a 3730XL DNA Analyzer (Applied Biosystems, Foster City, CA) at Genome Quebec (McGill University, Montreal, Quebec), or (2) a CEQ 8000 Genetic Analysis System (Beckman-Coulter, Fullerton, CA) at Queen's Ecology, Evolution and Behaviour Core Genotyping Facility (Queen's University, Kingston, Ontario).

**Table 2:** Sequence and location of all primers used to amplify parts of the mitochondrial genome

Primer	Location	Sequence	Source
b2	5' end of cytochrome *b*	5'-GCCCCTCAGAATGATATTTGTCCTCA-3'	1
b3	middle of cytochrome *b*	5'-GGACGAGGCTTTTACTACGGCTC-3'	2
b4	middle of cytochrome *b*	5'-TTGCTGGGGTGAAGTTTTCTGGGTC-3'	2
b5	3' end of cytochrome *b*	5'-TTCCACCCCTACTTCTCACTAAAAGA-3'	2
cytb-endL	3' end of cytochrome *b*	5'-TATCATCGGCCAACTAGCC-3'	3
b6	tRNA-Threonine	5'-GTCTTCAGTTTTTGGTTTACAAGAC-3'	2
16363H	5' end of ND6	5'-GTTGTGACCGTTGATAGTG-3'	3
ND616363	5' end of ND6	5'-CACTATCAACGGTCACAAC-3'	3
tGlu-boobyH	tRNA-Glutamic Acid	5'-ACAACGGCGGCATTTCAGGCC-3'	3
SsMCR-L143B	5' end of CR1	5'-ATTGCACATTARATTTASCT-3'	3
SsMCR-H170B	5' end of CR1	5'-ATGAAAGTATTATGTGATCC-3'	3
SlMCR-L160B	5' end of CR1	5'-ATCCACATTGCACATTAAGT-3'	3
SlMCR-H171B	5' end of CR1	5'-CATCAATTTACATATGTCGAC-3'	3
SdMCR-H750	middle of CR1 and CR2	5'-GGGAACCAAAAGAGGAAAACC-3'	4
SlMCR-L740	middle of CR1 and CR2	5'-GCATAGGAAGTACTTACAATCTAGG-3'	3
SsMCR-L143A	5' end of CR2	5'-ATTGCACATTARATTTAYTC-3'	3
SsMCR-L143A2	5' end of CR2	5'-ATTGCACATTAAATTTAGTC-3'	3
SsMCR-H169A	5' end of CR2	5'-TGAAAGTATACAGTCCATTG-3'	3
SlMCR-L160A	5' end of CR2	5'-ATCCACATTGCACATTTAAA-3'	3
SlMCR-H194A	5' end of CR2	5'-CAGTGGTATGTGTTTGTC-3'	3
12SH1301	5' end of 12SrRNA	5'-GGTAAGGTTAGGACTAAGTC-3'	3

Source refers to the original source of the primer sequence (1 = Kocher *et al. *[33], 2 = T. Birt *unpublished*, 3 = this study, 4 = Steeves *et al. *[34]). See Figure 1 for a graphical representation of primer locations.

Using this sequence as a guide, we next amplified the region located between the genes encoding for cytochrome *b *and 12SrRNA (i.e., the entire region spanning the duplicated area) in three overlapping fragments using the primer pairs b3/SlMCR-H171B, SlMCR-L160B/SlMCR-H194A and SlMCR-L160A/12SH1301 in one brown and one blue-footed booby (Figure 1). We amplified equivalent fragments in one red-footed booby using primer pairs b3/SsMCR-H170B, SsMCR-L143B/SsMCR-H169A and SsMCR-L143A/12SH1301 (Figure 1). We sequenced both strands of the PCR product using the original amplification primers and multiple internal primers (Figure 1, Table 2). As before, most regions were sequenced with at least two primers on each strand. This sequencing effort revealed a duplicated portion of the mtDNA genome that not only contained a second control region, but also involved copies of the genes for tRNA^Glu^, ND6, tRNA^Pro^, tRNA^Thr ^and a partial copy of cytochrome *b *(see Results for more details).

To further investigate the evolution of the duplicated regions, we focused our effort on domains I and II of the control region. Taking advantage of sequence differences in the 5' end of the duplicate control regions (which we termed CR1 and CR2), we used control region specific L-strand primers (i.e., primers that will only bind to one copy or the other) to amplify approximately 540 base pairs of CR1 and CR2 separately in 21 individuals of each species. Specifically, we used primers SlMCR-L160B and SlMCR-L160A paired with SdMCR-H750 to amplify CR1 and CR2, respectively, in brown and blue-footed boobies. In red-footed boobies we amplified CR1 with primers SsMCR-L143B and SdMCR-H750. We originally designed primer SsMCR-L143A as an L-strand primer to amplify CR2 in red-footed boobies (paired with SdMCR-H750), but due to a presumed mutation in the priming site in some individuals, we also designed another primer, SsMCR-L143A2, to amplify CR2 in some red-footed boobies. To verify that PCRs using SsMCR-L143A and SsMCR-L143A2 amplified homologous regions we compared the resultant sequence to sequence generated from long template PCR amplifications using a suite of different primers (not shown).

All PCR reactions were performed in 15 μL reactions under standard conditions (10 mM Tris pH 8.5, 50 mM KCl, 1.5 mM MgCl_2_, 1.6 μM bovine serum albumin, 2% gelatin, 0.2 mM each of the four dNTP's, 0.4 mM each of the heavy and light strand primers and 0.5 units of *Thermus aquaticus *[Taq] DNA polymerase [Quiagen, Mississauga]) with annealing temperature between 60 - 63°C. Both strands of the PCR product were sequenced with the amplification primers at Genome Quebec, as above.

Control region sequences were aligned using ClustalW [12]as implemented in BioEdit Version 7.0.5.3 [13]. Some control region sequences (both CR1 and CR2 sequences) had "ambiguous" sites where two nucleotides were present in the chromatogram (see Results). This ambiguity was also found in a large number (>100) of brown and red-footed booby CR2 sequences in a broader phylogeographic survey [14]. To eliminate the possibility that ambiguous sites were caused by sequencing errors, Morris-Pocock et al. [14] re-extracted and re-sequenced 20% of brown and red-footed booby individuals that had ambiguous sites: all sequences (including ambiguous sites) were identical. Additionally, a subset of individuals with sequence ambiguities in the current study were re-extracted and re-sequenced at the Queen's Ecology, Evolution and Behaviour Core Genotyping Facility, as above.

### Phylogenetic methods

We used both Bayesian inference and Maximum Likelihood (ML) approaches (as implemented in MrBayes Version 3.1.2 [15] and GARLI Version 0.95 [16] respectively) to estimate unrooted phylogenetic trees for CR1 and CR2 sequences from all 21 individuals from all three species. We used the default settings in GARLI and the nucleotide substitution model that best fit the data as determined by MrModeltest Version 2.2 [17], and evaluated reliability of the trees using 100 bootstrap replicates. In MrBayes we used one cold chain and three incrementally heated chains to explore parameter space, and ran each analysis for 10000000 generations, sampling every 100 generations. To verify that the Metropolis-coupled Markov chain Monte Carlo (MCMCMC) process was converging we ran two simultaneous runs and ensured that the MCMCMC process was continued until the standard deviation of split frequencies between the chains was lower than 0.01. To further ensure convergence, we re-ran all GARLI and MrBayes analyses three times. All results were consistent across runs and only one run is presented here.

## Results

### Confirmation of mitochondrial origin of sequences

A number of lines of evidence suggest that all regions sequenced are of mitochondrial origin, rather than nuclear pseudogenes [18]: (1) all putative tRNA sequences folded into the expected clover-leaf secondary structure [19]; (2) with the exception of a degenerate copy of the cytochrome *b *gene found in all species (see below), all protein coding genes appeared functional and contained no premature stop codons; (3) substitutions in protein coding genes between the three species were predominantly at the third codon position (see additional file 1); (4) regions with high similarity to avian conserved blocks were found in the expected locations in domain II (F, D and C Boxes, see additional file 2; [20]) and domain III (CSB-1; [20]) in both CR1 and CR2; (5) base pair composition of the L-strand was biased against Gs (26% C, 32% T, 27% A, 15% G); (6) variable sites were distributed as expected [20], with 185 in domain I and 37 in domain II (considering both CR1 and CR2); and (7) all regions were sequenced from long template PCR products, which seems to reduce the amplification of nuclear copies [21].

### Structure of the sulid mitochondrial genome

We obtained 5433, 5599 and 5457 base pairs of sequence from brown, red-footed and blue-footed boobies respectively. Results confirmed that the mitochondrial genomes of all three species contain a large fragment that has been duplicated (Figure 1). The observed gene order surrounding the duplicated area was identical to the albatross gene order [5] except that only a partial cytochrome *b *copy was found in boobies, whereas a partial copy and a separate, but degenerate cytochrome *b *copy was found in albatrosses. Remarkably, within species the duplicated regions have 100% sequence similarity, with the following three exceptions: (1) in all three species, one cytochrome *b *gene appeared functional, while the second copy was identical in sequence, but did not have the complete coding region and was presumably non-functional; (2) approximately 50 base pairs at the 5' end of the control regions differed significantly between CR1 and CR2; and (3) the 3' end of domain III in CR2 contained short repetitive sequence (CAAA) that was not detected in CR1. The size of the partial cytochrome *b *gene varied only slightly among the three species. Specifically, the degenerate gene was 546, 548, or 545 base pairs long in brown, red-footed and blue-footed boobies, respectively (see additional file 1). Both CR1 and CR2 consisted of a hypervariable domain I (containing a poly-cytosine repeat), a conserved domain II and a variable domain III containing complex repetitive sequence. The number of repeats in domain III in both brown and red-footed boobies appeared heteroplasmic within individuals and the repetitive sequences precluded complete sequencing of domain III in both CR1 and CR2 (Figure 1, grey areas). Because we were unable to cleanly sequence through these repetitive regions, we were unable to determine whether the number of repeats in CR1 and CR2 differed within species. Based on the size of electrophoresed PCR products on ethidium bromide stained gels, the length of CR1 (including the repetitive region) was approximately 2700, 1700, and 1500 bp in brown, red-footed, and blue-footed boobies, respectively. The length of CR2 was approximately 2100 bp in brown boobies and 1600 bp in both red-footed and blue-footed boobies.

### Mitochondrial single site heteroplasmy

Of the 126 control regions sequenced (2 copies in each of 63 individuals), 28 contained one or two "ambiguous sites": sites in the chromatogram with two overlapping peaks of which the second base was at least 25% of the height of the called base. Ambiguous sites were found in both CR1 and CR2, and in all three species. Importantly, these ambiguous sites differed from ambiguous sites resulting from co-amplification of CR1 and CR2. Specifically, we could rule out the co-amplification of CR1 and CR2 because this would result in multiple ambiguous sites (>10), all located at the 5' end of the sequence (see below). In addition, LT-PCR amplification and sequencing with multiple internal primers verified an ambiguous site in CR1 of red-footed booby individual rf_CocD7, and re-sequencing of a subset of individuals with ambiguities verified our original results. We can also reasonably eliminate the possibility that these ambiguities are the result of co-amplification of nuclear pseudogenes for the reasons outlined in the section above. We therefore inferred that they represent true mitochondrial heteroplasmy.

### Evolution of the sulid control region

We found 116 unique haplotypes in the 126 control regions that we sequenced (sequences are deposited in Genbank, accession numbers GU290353 - GU290478). No haplotypes were shared either between species or between CR1 and CR2 within a species. Inspection of sequence alignments revealed a striking pattern of variation when considering CR1 and CR2 within individuals. With the exception of approximately the first 50 base pairs of sequence, CR1 and CR2 sequences were identical or differed by only one or two mutations within all individuals. In contrast, the first approximately 50 base pairs at the 5' end of domain I were extremely divergent within individuals. Preliminary analyses also revealed that phylogenetic signals from the first 50 base pairs alone and the remaining base pairs alone were conflicting (see below for more details). A common test used to detect discordant phylogenetic signal within DNA sequence data is the 4-gamete test [22]; however, this test assumes an infinite sites model that is probably not appropriate for hyper-variable control region data. Therefore, we inspected the sequence alignment and visually split all control region sequences into a 5' "variable" section (the first 39 - 49 bp) and a 3' "conserved" section (the remaining 493 base pairs). We were confident in our assignment of the break-point between the 5' and 3' sections because CR1 and CR2 sequences were either identical or differed by a maximum of two mutations downstream of the breakpoint. We performed all subsequent analyses independently on the 5' and 3' data sets.

**Figure 2:**
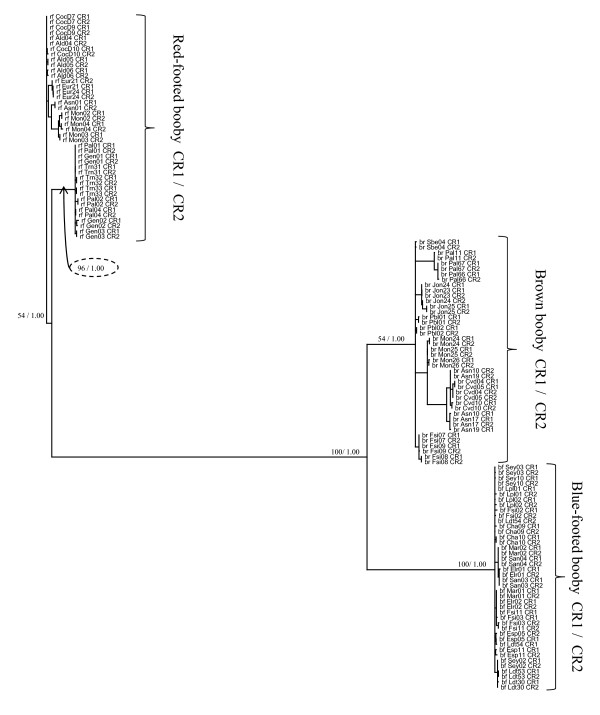
**Maximum likelihood phylogenetic trees for the 3' end of CR1 and CR2 sequences**. Unrooted maximum likelihood phylogenetic tree for the 3' end of CR1 and CR2 sequences from 21 brown, red-footed and blue footed boobies each. Nodal support of major clades is shown first for 100 maximum likelihood bootstrap pseudo-replicates and then for Bayesian posterior probability.

**Figure 3:**
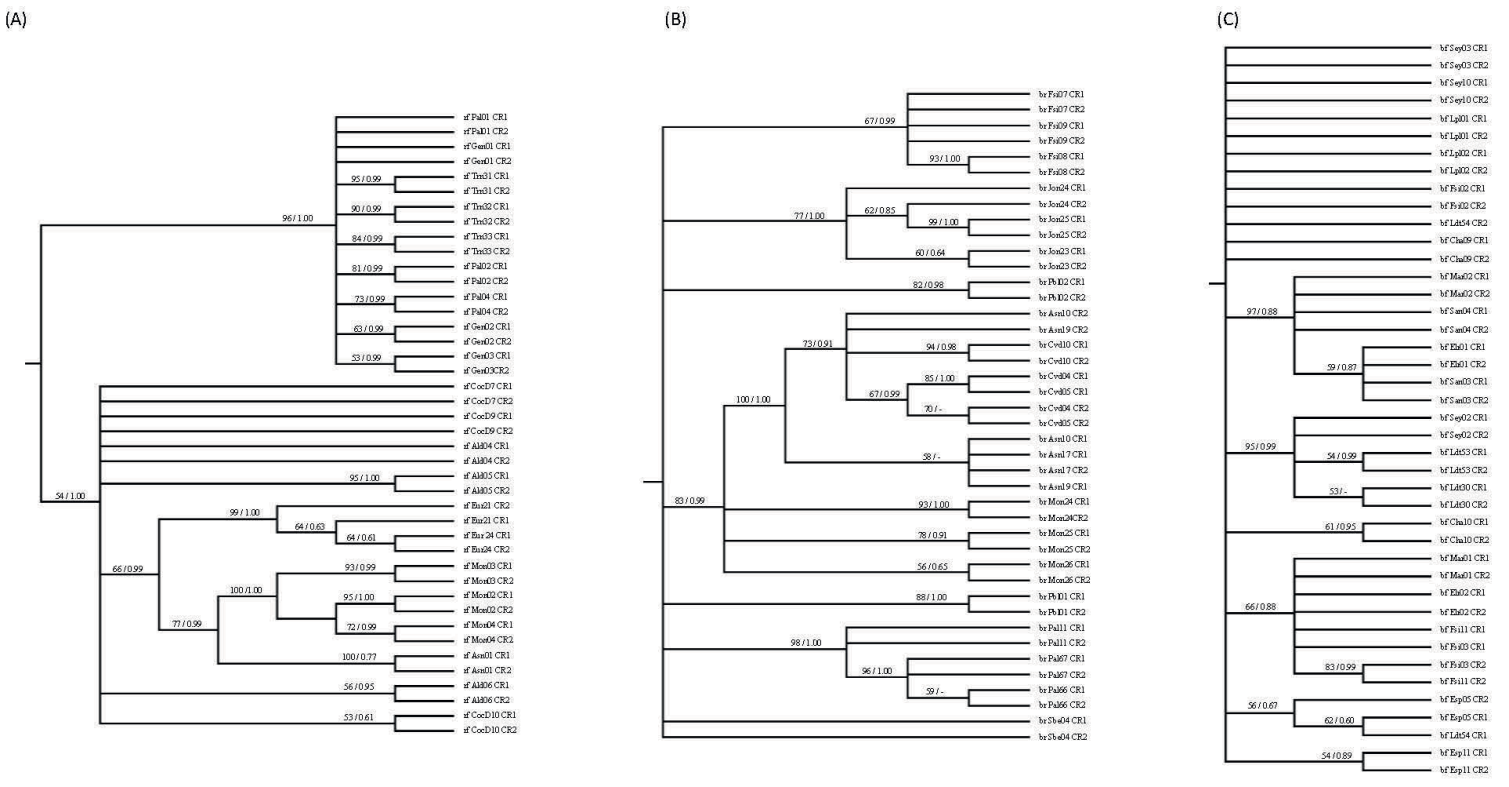
**Maximum likelihood cladograms for the 3' end of CR1 and CR2 sequences**. Unrooted maximum likelihood phylogenetic trees for the 3' end of CR1 and CR2 for (a) brown, (b) blue-footed, and (c) red-footed boobies (shown as cladograms to make support values easier to read).

For the 3' data set, the most likely model of nucleotide substitution as suggested both by hierarchical likelihood tests and by AIC values in MrModeltest was the Hasegawa-Kishino-Yano model [23] with a proportion of invariant sites and gamma distributed rate variation (HKY+I+G). Trees estimated using maximum likelihood and Bayesian phylogenetic inference were similar and all major clades were recovered by both methods. Bayesian inference resolved many more recent clades (albeit with low posterior probability) while ML collapsed many of these clades into polytomies. We have presented the maximum likelihood tree for the 3' data set and indicated support for clades with both bootstrap values (from the ML analysis) and posterior probabilities (from the Bayesian analysis) on the maximum likelihood tree (Figure 2, Figure 3). CR1 and CR2 haplotypes from a given species grouped into a well supported monophyletic clade for each of the three species (Figure 2). Investigating the topology of each major clade in more detail revealed that often CR1 and CR2 haplotypes from single individuals form clades exclusive of all other haplotypes (Figure 3). In other words, paralogous control regions are often more closely related than orthologous control regions, often with very high bootstrap support and Bayesian posterior probability. Moreover, the average sequence divergence between paralogous control regions within individuals was 0.2%, while the average sequence divergence between orthologous control regions within a population (e.g., between CR1 sequences found in individuals from the same species at the same colony) was 1.1%.

For the 5' data set, we were unable to reliably align all CR1 and CR2 haplotypes due to the high variability and short sequence length (see additional file 2). However, a number of characteristics of the sequence data suggest that the phylogenetic signal from the 5' data set differed fundamentally from the 3' data set signal. First, sequences from CR1 alone and CR2 alone were easily aligned within species, but could not be aligned to each other (e.g., all brown booby CR1 sequences could be aligned to each other with no gaps, but could not be aligned to any brown booby CR2 sequence). Second, brown booby CR1 sequences were easily aligned to blue-footed booby CR1 sequences. Similarly, brown and blue-footed booby CR2 sequences were easily aligned. Overall, for the approximately 50 base pairs at the 5' end of the control region, orthologous sequences appeared to be more closely related than paralogous sequences.

## Discussion

The most interesting result from the present study is that duplicated regions of the mitochondrial genomes of three seabird species are evolving in concert. We found a large duplication in part of the mtDNA genome that was present and structurally identical in all three species. Molecular phylogenetic analyses suggest that brown, red-footed and blue-footed boobies diverged approximately three million years ago (MYA; [24]). Therefore, the duplication event likely occurred sometime before 3 MYA, although denser sampling of other closely related species is needed to pinpoint the exact timing of the duplication. Alternatively, gene duplication could have arisen multiple times within the booby lineage. Although the relative frequency of gene duplication within avian mtDNA has been debated in recent years [3,6,25], the similarity of the duplicated regions in the three booby species seems to preclude repeated convergent duplications within the Sulidae. Perhaps the most convincing argument for a single origin of the duplicated region in the Sulidae is that the partial cytochrome *b *copy differs in length by only three base pairs in the three species, and we find it unlikely that this pattern could emerge by parallel duplication and degeneracy. Importantly, the duplicated mtDNA region documented in our study includes the mitochondrial control region in all three species.

Further investigation of the evolution of duplicate control regions indicated that paralogous control regions were often more closely related than orthologous control regions. The pattern of concerted evolution documented in the current study is, perhaps, most similar to the pattern found in mangrove killifish [7] and *Thalassarche *albatrosses [5]. Specifically, Tatarenkov and Avise [7] found that duplicate control regions within a single individual were either identical (77 individuals) or differed by only a single substitution (11 individuals). While we documented similar concerted evolution in the majority of the control region, we detected a discordant phylogenetic signal at the 5' end of the control region, which appeared to be evolving independently. Abbott et al. [5] found similarly conflicting phylogenetic patterns in 5' and 3' segments of duplicated control regions in *Thalassarche *albatrosses, however their study did not have the dense population-level sampling equivalent to Tatarenkov and Avise [7], and thus they were unable to comment on the rate of concerted evolution (i.e., how often the 3' segments of the two control regions were homogenized). To our knowledge, our study is the first to document both the discordant phylogenetic signal mentioned above, and the rapid pace of concerted evolution of the 3' segment using a large sample of conspecific individuals.

Our study also found evidence that regions adjacent to the control region are evolving in concert. Although we sequenced the entire mtDNA duplication in only one individual of each species (3 individuals total), this sequence suggests that tRNA^Thr^, tRNA^Pro^, ND6, tRNA^Glu^, and part of cytochrome *b *may also be evolving in concert. Specifically, the duplicated tRNA^Thr^, tRNA^Pro^, ND6, tRNA^Glu ^sequences, the portion of cytochrome *b *that was duplicated, and all duplicated intergenic nucleotides were identical within each species, while the sequences differed by up to 12% between species (see additional file 1). If these regions had been evolving independently we would expect, for example, that sequence from orthologous ND6 sequences would be more closely related across species than paralogous ND6 sequences within individuals. To further verify that concerted evolution is also occurring in these segments, the entire duplicated region (cytochrome *b *- 12S rRNA) should be amplified in a large number of individuals as was done for the control region in this study. Abbott et al. [5] also suggest that ND6 may be evolving in concert in *Thalassarche *albatrosses, but similarly acknowledge that this conclusion is tentative pending further individual sampling. In both albatrosses and boobies, the emerging pattern is that a large segment of mtDNA stretching from tRNA^Thr ^to the control region is evolving in concert, while a smaller region nested within this fragment either is evolving entirely independently, or in concert, albeit with a slower rate of homogenization.

The exact molecular mechanism that facilitates concerted evolution of animal mtDNA is unknown, but has most often been attributed to frequent tandem gene duplication and elimination due to slipped-strand mispairing during DNA replication, gene conversion via crossing over of nicked strands or parallel selection on duplicated regions [4,26]. Parallel selection acting on duplicated regions can potentially explain concerted evolution of functional mtDNA regions (e.g., ND6, avian conserved boxes in domain II of the control region), but it cannot account for the observed concerted evolution of presumably non-functional parts of the control region. Eberhard et al. [4] suggest that intra-molecular recombination may occur between parental and nascent strands of duplicated control regions during DNA replication when DNA is three-stranded, resulting in concerted evolution via gene conversion. Specifically, the nascent H-strand of one control region may recombine with the homologous parental strand of the other control region, leading to the homogenization of both sequences. This mechanism can adequately explain the concerted evolution of the control region, but does not obviously account for the concerted evolution of regions upstream of the control region (tRNAs, ND6). Our data are consistent with the gene conversion model outlined by Kumazawa et al. [26]; however, similar to *Thalassarche *albatrosses, multiple recombination points are needed to explain the non-concerted evolution of the 5' section of the control region [5]. Ultimately, the exact molecular mechanism underlying concerted evolution of mtDNA may be difficult to identify using a phylogenetic approach.

## Conclusions

The use of mtDNA in phylogeographic studies has recently been criticized for a number of reasons (e.g., [27]). Despite these shortcomings, mitochondrial DNA still has a foothold in phylogeography and is potentially very informative if used appropriately [28]. Proponents of mtDNA cite a number of advantages over other molecular markers including a fast evolutionary rate, a small effective population size (approximately 1/4 that of nuclear DNA), rare heteroplasmy and an apparent lack of recombination [28,29]. Recently, however, some of these fundamental tenets have been challenged. Perhaps most interestingly, mtDNA recombination has been documented in a range of taxa including sea cucumbers, snakes, ticks, birds, marine ostracods and fish [5,7,26,30-32].

We demonstrated that concerted evolution of duplicated control regions occurs in at least three species within the Sulidae. Perhaps more interesting is that a very similar mtDNA duplication and concerted evolution pattern is found in *Thalassarche *albatrosses. The Sulidae and albatrosses are part of a large clade that also contains all procellariiform and most pelecaniform seabirds [8]. Therefore, concerted evolution must either be extremely widespread and undocumented within this lineage or have arisen many times.

The extent that concerted evolution can affect phylogeographic studies depends on the pace of gene conversion relative to the nucleotide substitution rate. If gene conversion occurs less often than mutation, specific care must be taken to amplify homologous control regions in a population genetic study. Fortunately, because paralogous control regions within individuals are often identical, the pace of gene conversion in boobies appears to be so rapid that amplification of homologous regions should be less of a problem. However, because concerted evolution of duplicated control regions may be widespread, we recommend that caution should be exercised when using the control region as a molecular marker in population genetic or phylogenetic analyses. Moreover, because separate parts of a single control region may be evolving differently, we strongly urge researchers to test for conflicting phylogenetic signals in the control region before using mtDNA sequence variation for further analyses. We also suggest that future work should expand the taxonomic scope of the present study. Preliminary evidence indicated that concerted evolution also occurs in at least four other sulid species (northern gannet *Morus bassanus*, Birt T, unpublished data; masked booby *S. dactylatra *and Nazca booby *S. granti*, Steeves TE, personal communication, and Peruvian booby *S. variegata*, Taylor S, unpublished data), however the pattern should also be investigated in other genera in the seabird clade (e.g., pelicans, frigatebirds, penguins; [8]) to further test whether concerted evolution of duplicate control regions evolved once, or multiple times in parallel, among seabirds.

## Authors' contributions

JAMP designed the study, conducted long PCR lab work and control region specific lab work, performed genetic analysis and wrote the manuscript. SAT helped design the study, performed control region specific lab work and critically revised the manuscript several times. TPB helped design the study and critically revised the manuscript several times. VLF helped design the study, supervised data analysis and critically revised the manuscript several times. All authors read and approved the final paper.

## Supplementary Material

Additional file 1 **Nucleotide sequence of duplicated mitochondrial regions**. Sequence of the duplicated (a) cytochrome *b*, (b) tRNA^Thr^, (c) tRNA^Pro^, (d) tRNA^Glu^, and (e) ND6 genes from one red-footed (RF), brown (BR), and blue-footed booby (BF) each. Identity to the red-footed booby sequence is shown with asterisks. RF2, BR2 and BF2 refer to the 2^nd ^copy of the gene in each species (see Figure 1). *p*RF, *p*BR and *p*BF represent partial cytochrome *b *copies in each species. Dashes represent bases that were not found in the partial copy. Anticodons in tRNA genes are underlined. Click here for file

Additional file 2 **Nucleotide sequence of CR1 and CR2**. Sequence of CR1 and CR2 from one red-footed (RF), brown (BR), and blue-footed booby (BF) each. Identity to the red-footed booby CR1 sequence is shown with asterisks. Dashes represent indel polymorphisms. The grey box represents the 5' variable section that could not be easily aligned. Avian conserved sequence blocks F, D, and C (Baker and Marshall 1997) are underlined. Note that only domains I and II of the control region are shown. Click here for file
