# Supplementary material for: Concerted evolution of duplicated mitochondrial control regions in three related seabird species
Source: BMC Evol Biol. 2010 Jan 14;10:14. doi: 10.1186/1471-2148-10-14 (PMC2820450; doi:10.1186/1471-2148-10-14)
Supplement: Additional file 1 — Nucleotide sequence of duplicated mitochondrial regions. Sequence of the duplicated (a) cytochrome b, (b) tRNAThr, (c) tRNAPro, (d) tRNAGlu, and (e) ND6 genes from one red-footed (RF), brown (BR), and blue-footed booby (BF) each. Identity to the red-footed booby sequence is shown with asterisks. RF2, BR2 and BF2 refer to the 2nd copy of the gene in each species (see Figure 1). pRF, pBR and pBF represent partial cytochrome b copies in each species. Dashes represent bases that were not found in the partial copy. Anticodons in tRNA genes are underlined. [file 1471-2148-10-14-S1.PDF]

Fig S1 Sequence of the duplicated (a) cytochrome *b*, (b) tRNA^{Thr}, (c) tRNA^{Pro}, (d) tRNA^{Glu}, and (e) ND6 genes from one red-footed (RF), brown (BR), and blue-footed booby (BF) each. Identity to the red-footed booby sequence is shown with asterisks. RF2, BR2 and BF2 refer to the 2nd copy of the gene in each species (see Figure 1). *p*RF, *p*BR and *p*BF represent partial cytochrome *b* copies in each species. Dashes represent bases that were not found in the partial copy. Anticodons in tRNA genes are underlined.

(a)

```
RF : ACAGGAGTAATTCTCCTACTGACACTAATAGCAACTGCCTTCGTAGGCTATGTCCTACCATGAGGACAAATATCCTTCTG 80
pRF: --------------------------------------------------------------------------------
BR : ********************A********************************T**G***********************
pBR: --------------------------------------------------------------------------------
BF : ***********C********A*****T*****************************G***********************
pBF :--------------------------------------------------------------------------------

RF : AGGAGCCACAGTCATTACCAACCTATTCTCAGCCATTCCATACATTGGCCAAACCCTCGTAGAATGAGCTTGAGGCGGAT 160
pRF: --------------------------------------------------------------------------------
BR : ***************C******T*******G*****C**C**************A*************************
pBR: --------------------------------------------------------------------------------
BF : ***************C******T*******G*****C*****************A*************************
pBF: --------------------------------------------------------------------------------

RF : TTTCAGTAGACAACCCTACCCTAACTCGATTCTTTGCTCTACACTTCCTCCTCCCATTCATTATCGCAGGTCTCGTCCTA 240
pRF: --------------------------------------------------------------------------------
BR : *C**C*****T*****C********C********CA*C**************************T***A*C**T******
pBR: --------------------------------------------------------------------------------
BF : *C********T**T***********C********C**C*****T********************T*****C**T*****G
pBF: --------------------------------------------------------------------------------

RF : ATCCATCTCACATTCCTCCACGAATCAGGCTCAAACAACCCACTAGGAATCTCATCAAACTCCGACAAAATCCCATTCCA 320
pRF: ----------**********************************************************************
BR : G*T***********************G********T***********C**************T*****************
pBR: ------------**************G********T***********C**************T*****************
BF : *****C*****C***********************************C**************T**************T**
pBF: -------------**********************************C**************T**************T**
```

```
RF : CCCCTACTTCACCCTAAAAGACATACTAGGATTCATACTCCTTCTACTTCCACTAACAGCCATAGCCCTATTCTCCCCCA 400
pRF: ********************************************************************************
BR : ***********************C*********T******A*G***************A*********************
pBR: ***********************C*********T******A*G***************A*********************
BF : ******T**************TG**********T******A*A***************A*********************
pBF: ******T**************TG**********T******A*A***************A*********************

RF : ACCTCCTCGGAGATCCAGAAAACTTCACCCCAGCAAACCCCCTCGTCACTCCCCCCCACATTAAACCAGAATGGTACTTC 480
pRF: ********************************************************************************
BR : *******T*****C***********************T**T********C**A***********G***************
pBR: *******T*****C***********************T**T********C**A***********G***************
BF : *T*****T*****C**************************T********C**A*****************G*********
pBF: *T*****T*****C**************************T********C**A*****************G*********

RF : CTATTCGCATACGCCATTCTACGCTCAATCCCAAACAAACTAGGAGGAGTACTAGCCCTAGCAGCCTCCGTCCTAATCCT 560
pRF: ********************************************************************************
BR : ***********T********************C***********G***********************************
pBR: ***********T********************C***********G***********************************
BF : **G**************************T**C***********************************************
pBF: **G**************************T**C***********************************************

RF : CTTCCTCAGCCCCTTCCTCCACAAATCCAAACAACGTACAATAACCTTCCGTCCCCTCTCCCAACTCCTATTCTGAACCT 640
pRF: ********************************************************************************
BR : ***************T********************************T**C********T**************G**TC
pBR: ***************T********************************T**C********T**************G**TC
BF : ***************T********************************************T**************AG**C
pBF: ***************T********************************************T**************AG**C
```

```
RF : TAGTCGCCAACCTCCTCATCCTAACATGAGTTGGTAGCCAACCTGTAGAGCACCCATTCATTATCATCGGCCAACTAGCC 720
pRF: ********************************************************************************
BR : *************A*****T***********C**C**************A******************************
pBR: *************A*****T***********C**C**************A******************************
BF : **********T********T***********C**C**************A*****************T********T***
pBF: **********T********T***********C**C**************A*****************T********T***

RF : TCCCTTACCTACTTCACTATCCTCCTCATCCTCTTCCCCCTTATCGGAGCCCTAGAAAATAAAATACTCAATTACTAA 798
pRF: ******************************************************************************
BR : *****C**T*****T**C*****************T*****C*****G***********C***********C******
pBR: *****C**T*****T**C*****************T*****C*****G***********C***********C******
BF : *****C***********C*****************T*****C*****************C***********C******
pBF: *****C***********C*****************T*****C*****************C***********C******
```

(b)

```
RF : CTCTAATAGTTTACAAAAAACATTGGTCTTGTAAACCAAAGAATGAAGGCTACATCCCTTCTTAGAGTT 69
RF2: *********************************************************************
BR : ***************G******C*******************GC**********C**************
BR2: ***************G******C*******************GC**********C**************
BF : ******************************************GC*****T*G**C**************
BF2: ******************************************GC*****T*G**C**************
```

(c)

```
RF : TCAGAGAAAAAGGGTTCAAACCTTCACCTCCAGCTCCCAAAGCTGATATTCTTCATTGAACTATCCTCTG 70
RF2: **********************************************************************
BR : ********G****AC*************CT**********************A****A************
BR2: ********G****AC*************CT**********************A****A************
BF : ********G****AC**************T**********************A****A************
BF2: ********G****AC**************T**********************A****A************
```

(d)

```
RF : CCTAACCGCCCGAATCGCCCCCCGCGACAACCCCCGTACAAGCTCCAACACAACAAACAAAGTCAACAACAACCCTCACC 80
RF2: ********************************************************************************
BR : *TA***T********A**T*****************C********T**********************************
BR2: *TA***T********A**T*****************C********T**********************************
BF : *TA***T**T*****A*****************T**C********C*************************G********
BF2: *TA***T**T*****A*****************T**C********C*************************G********

RF : CAGCCAACACAAATAACCCCGCCCCCTGAGAATAAAACATCGCTACACCACCAAAATCCAACCGAACTAAAAACATCCCT 160
RF2: ********************************************************************************
BR : *******T*****C*****T*****T**G***********A*****G****T*********T*****C*******CT**C
BR2: *******T*****C*****T*****T**G***********A*****G****T*********T*****C*******CT**C
BF : *************C*****T********G***********A*****G****T******T**T*****C*****T*CT**C
BF2: *************C*****T********G***********A*****G****T******T**T*****C*****T*CT**C

RF : CCACTATCAACAGTCACAACCCCTAACTTCCATCCTTCCACCAACCCACCAATCACAACCCCAACCCCAAGCACTAAAAT 240
RF2: ********************************************************************************
BR : ***********G********************C**C*************T***AG**C*********************C
BR2: ***********G********************C**C*************T***AG**C*********************C
BF : ***********A********************C**C*************T***AG*GG****************C****C
BF2: ***********A********************C**C*************T***AG*GG****************C****C

RF : AAACCCCACCCCATACCCCACAACCCCTCAATTATCCCAGGCTTCCGGAAACGGATCCGCAGCTAAAGCCACAGAGTATA 320
RF2: ********************************************************************************
BR : **GT**************TG************C***T**AC*C**A*****************C*****T********C*
BR2: **GT**************TG************C***T**AC*C**A*****************C*****T********C*
BF : ******************TG*******C****C***T**AG*C**A*****************C**************C*
BF2: ******************TG*******C****C***T**AG*C**A*****************C**************C*
```

```
RF : CAAAAACCACCAACATCCCCCCCAAATAGACCATAAATAGTACCAACGCCACAAAAGACACCCCCAAACTCAACAACCAC 400
RF2: ********************************************************************************
BR : ****************A**T*****G**C********C*AA***********************************T***
BR2: ****************A**T*****G**C********C*AA***********************************T***
BF : ****************G*****T*****C********C*AA****G******************************T***
BF2: ****************G*****T*****C********C*AA****G******************************T***
```

```
RF : CCGCATCCCGCCACAGACGCTAGCACCAACCCTACCACCCCATAATAAGGTGCAGGATTAGACCCAACCGCCAAAGCACC 480
RF2: ********************************************************************************
BR : **A**C**T********T****AT*****A**C*****************************AG****T***********
BR2: **A**C**T********T****AT*****A**C*****************************AG****T***********
BF : **A**C****************AT*****A**C*****************C***********AG****T***********
BF2: **A**C****************AT*****A**C*****************C***********AG****T***********
```

```
RF : TAAAACAAAGCACACCCCCAAAACAATTACAAAGTAAGTCAT 522
RF2: ******************************************
BR : ************T*********G***C***************
BR2: ************T*********G***C***************
BF : C********A************G*******************
BF2: C********A************G*******************
```

(e)

```
RF : AGTTCCTGCTTGGCCTCTCTCCAAGCTATGCGGCCTGAAATGCCGCCGTTGTAAACCTCAACCACAGGAAC 71
RF2: ***********************************************************************
BR : ***********************************************************************
BR2: ***********************************************************************
BF : ***************************G*******************************************
BF2: ***************************G*******************************************
```
