# Supplementary material for: Concerted evolution of duplicated mitochondrial control regions in three related seabird species
Source: BMC Evol Biol. 2010 Jan 14;10:14. doi: 10.1186/1471-2148-10-14 (PMC2820450; doi:10.1186/1471-2148-10-14)
Supplement: Additional file 2 — Nucleotide sequence of CR1 and CR2. Sequence of CR1 and CR2 from one red-footed (RF), brown (BR), and blue-footed booby (BF) each. Identity to the red-footed booby CR1 sequence is shown with asterisks. Dashes represent indel polymorphisms. The grey box represents the 5' variable section that could not be easily aligned. Avian conserved sequence blocks F, D, and C (Baker and Marshall 1997) are underlined. Note that only domains I and II of the control region are shown. [file 1471-2148-10-14-S2.PDF]

Fig S2 Sequence of CR1 and CR2 from one red-footed (RF), brown (BR), and blue-footed booby (BF) each. Identity to the red-footed booby CR1 sequence is shown with asterisks. Dashes represent indel polymorphisms. The grey box represents the 5’ variable section that could not be easily aligned. Avian conserved sequence blocks F, D, and C (Baker and Marshall 1997) are underlined. Note that only domains I and II of the control region are shown.

```
RF–CR1: CTAGAAAGCCATATAATTCATGTAAGGATCACATAATACTTTCATG   TTTTCAACACATTACCATACCCTTCGGATAG 80
RF-CR2: CTAGATTGATATTCACAATTCATGTACAACGGACTGTATACTTTCATG *******************************
BR-CR1: CTAAGTCGACATACGTAAATTCATGTATGAAGGCTATCTCTATCATGT -G*CT*G****G*TT*T**AA*CC******A
BR-CR2: CTAAAAAGACATGTCAATTCATGGAAACAAACACATTTCTATCATGTGT-G*CT*G****G*TT*T**AA*CC******A
BF-CR1: CTAAGTCGACATACAAAATCCATGTATGAAGATTATCTTTCCATGA   -ACCT******A*C*T***AA*C*T**GC*A
BF-CR2: CTAGAAAGGCATGACAATTCATGGAAACAATCGCATCAA          -ACCT******A*C*T***AA*C*T**GC*A

RF–CR1: TAACCTA-TACCTCCCCTGCCAAACCTACTGACTAACTCTGTAATGCTCCTCCATCAGCAGAATTATCT----CTACCTT 160
RF–CR2: ********************************************************************************
BR–CR1: G*C*T*T-*AT********GT*****C*ACC*T****C***********TAT*--**A*****CCC*T**CTA**C****
BR–CR2: G*C***T-*AT********GT*****C*ACC*T****C***********TAT*--**A*****CCC*T**CTA**C****
BF–CR1: *CC*TCTGA**********A*****************************TAT*--**A***CTACC*TC-CAG**CT*C*
BF–CR2: *CC*TCTGA**********A*****************************TAT*--**A***CTACC*TC-CAG**CT*C*

RF–CR1: ACCCAAACCATTCCAA---AACAATCCTCTGTACAAGCTTCAAACTTTCCAGGATACGGAAGTG--TAACAGAACCAAAC 240
RF–CR2: ********************************************************************************
BR–CR1: ***********AA*CTATCCT*G*--*C****G***A*C*****T*CCT***************GCC*C-******C*C*
BR–CR2: ***********AA*CTATCCT*G*--*C****G***A*C*****T*CCT***************GCC*C-******C*C*
BF–CR1: ***********GA*CT*TGCT**----CT*A***CTCTC*****T**CG***************GC**C***G**A**CT
BF–CR2: ***********GA*CT*TGCT**----CT*A***CTCTC*****T**CG***************GC**C***G**A**CT

RF–CR1: C--TGCAATGGTAACAAGACATACCCTCTCAACTCACTCTCGAAGTACCGGTTTCTGAAGAACCAGGTTATCTATTAATC 320
RF–CR2: ********************************************************************************
BR–CR1: *AG**A*********C**G****A**********-G********A*G**A**********G*TT************G***
BR–CR2: *AG**A*********C**G****A**********-G********A*G**A**********G*TT************G***
BF–CR1: TCG**A*****************G**********-CT*********G****C********G*TT************G***
BF–CR2: TCG**A*****************G**********-CT*********G****C********G*TT************G***
```

```
RF-CR1: GGATTTCTCACGTGAAATCAGCAACGCACCGCACGAAAGATCCTACGTTACTAGCTTCAGGACCATTCATTCCCCCTACA 400
RF-CR2: ********************************************************************************
BR-CR1: *AGC***********************G*******G********G***********************************
BR-CR2: *AGC***********************G*******G********G***********************************
BF-CR1: *AGC***********************G******A*********************************************
BF-CR2: *AGC***********************G******A*********************************************

RF-CR1: CCCCTAGCCCAACTTGCTCTTTTGCGCCTCTGGTTCCTCGGTCAGGGCCATAACTCGATTGATATAG-CACTCCTCACTT 480
RF-CR2: ********************************************************************************
BR-CR1: ********A*************************************A****CC*CT****A**TC*AATGT*****G*C*
BR-CR2: ********A*************************************A****CC*CT****A**TC*AATGT*****G*C*
BF-CR1: ********A*************************************A****CC*CT****A*CTC*AATGT*T*****C*
BF-CR2: ********A*************************************A****CC*CT****A*CTC*AATGT*T*****C*

RF-CR1: TTCACAGAGTCATCTGGTAGGCTATTTATCAT-CATTCTCCCTCTTAATCGCGTCACCGTAA 542
RF-CR2: **************************************************************
BR-CR1: *******************T*******G***AA*************G***************
BR-CR2: *******************T*******G***AA*************G***************
BF-CR1: *******************T***********A-*T***************************
BF-CR2: *******************T***********A-*T***************************
```
